# Associations between Movement Behaviours and Obesity Markers among Preschoolers Compliant and Non-Compliant with Sleep Duration: A Latent Profile Analysis

**DOI:** 10.3390/ijerph18189492

**Published:** 2021-09-08

**Authors:** Alesandra A. de Souza, Jorge A. P. S. Mota, Gustavo M. G. da Silva, Rafael M. Tassitano, Cain C. T. Clark, Michael J. Duncan, Clarice M. de L. Martins

**Affiliations:** 1 Department of Physical Education, Federal University of Tocantins, Tocantinopolis 77900-000, Brazil; 2 Research Centre of Physical Activity, Health and Leisure, Faculty of Sport Sciences, University of Porto, 4050-313 Porto, Portugal; jmota@fade.up.pt (J.A.P.S.M.); claricemartinsufpb@gmail.com (C.M.d.L.M.); 3 Laboratory for Integrative and Translational Research in Population Health (ITR), 4050-091 Porto, Portugal; 4 Research Centre in Sports Sciences, Health Sciences and Human Development (CIDESD), University of Maia (ISMAI), 4475-690 Maia, Portugal; gugonsilva@gmail.com; 5 Department of Physical Education, Federal Rural University of Pernambuco, Recife 50010-000, Brazil; rafael.tassitano@ufrpe.br; 6 Faculty of Health and Life Sciences, Coventry University, Coventry CV1 5FB, UK; ad0183@coventry.ac.uk; 7 Centre for Applied Biological and Exercise Sciences, Coventry University, Coventry CV1 5FB, UK; aa8396@coventry.ac.uk

**Keywords:** cluster analysis, physical activity, sedentary behaviour, obesity

## Abstract

This study identifies physical activity (PA) and sedentary behaviour (SB) clusters in preschoolers compliant (C) or non-compliant (NC) with sleep recommendations; and associates these clusters with obesity markers. PA and SB were objectively assessed (Actigraph WGT3-X) in 272 preschoolers (4.4 ± 0.7 years old). Sleep duration was parent-reported, and preschoolers were classified as C (3–4 years old: 600–780 min/day; 5 years old: 540–660 min/day) or NC with sleep recommendations. Body mass index (BMI) and waist circumference (WC) were assessed according to international protocols. Moderate to vigorous physical activity (MVPA) and light physical activity (LPA) were categorized as low/high (<60 min/>60 min/day or <180 min/180 min/day, respectively). SB was defined according to mean values between clusters. Latent profile analysis was performed. Associations between the observed clusters and obesity markers were determined using linear regression (RStudio; 1.3.1073). Four cluster solutions for C and NC preschoolers were identified. A negative association between C/Low MVPA cluster and BMI, and a positive association between NC/Low MVPA and BMI (β = −0.8, 95%CI = −1.6;−0.1, and β = 0.9, 95%CI = 0.1;1.7, respectively) were observed. No association was seen for SB clusters. Adequate sleep duration may have a protective role for preschoolers’ BMI, even if the children do not comply with MVPA recommendations.

## 1. Introduction

Sleep is a daily behaviour that positively or negatively impacts on the entire day [1], as short sleep duration has been associated with low emotional regulation [2] and cognitive function [3], and higher odds of obesity development when compared to adequate sleep duration [4]. Preschoolers with adequate sleep duration show lower rates of obesity [5,6] and better social and emotional health [7], compared to those who do not accrue adequate sleep. Therefore, the American Academy of Sleep Medicine [8] and Andersen et al. [9] recommend that parents/guardians adopt hygiene habits related to sleep, namely: having a comfortable, quiet, and dark room; maintaining regular hours for sleeping and waking up; avoiding exposing preschoolers to screens or caffeine intake before bed; regulating the number of hours of sedentary behaviour (SB) throughout the day, in addition to facilitating a physically active lifestyle [9,10].

The World Health Organization [11] recommends that children aged three to four years accumulate between 10 and 13 daily sleeping hours, while the American Academy of Sleep Medicine [8] suggests the same amount for three- to five-year-old children. Although there are specific recommendations for sleep duration among preschoolers, it is known that daily behaviours, such as sleep, physical activity (PA), and sedentary behaviour (SB), are co-dependent [12] and may cluster in different ways [13].

Of note, Latent Profile Analysis has been used to identify those with similar behaviours patterns, grouping them into classes [14]. Previous studies on children have used latent profile analysis to characterize sleep, PA, and SB cluster, for example [15,16], focusing on obesogenic-related outcomes [17,18,19,20,21].

In early childhood, Saldanha-Gomes et al. [20] explored the association between latent patterns of diet, sleep, PA, and SB with obesity markers, and showed that five-year-old girls allocated to the high TV screen time/high outdoor PA cluster had 10% more body fat those in other clusters. This study highlighted important evidence in the current literature, exploring latent patterns of movement behaviours and obesity markers in preschoolers. However, sleep duration varies substantially, and there is great variability in sleep patterns between preschool age children [22,23]. Moreover, the association between clusters of movement behaviours and obesity markers, such as body index mass (BMI) and waist circumference (WC), between those children who comply or do not comply with sleep recommendations remains an unexplored issue.

Results from this specific age group are limited, even considering that several health-related risks factors appear in this period of life [24,25,26], and behaviours adopted in early childhood tend to track throughout life [27]. Therefore, this study sought to identify PA and SB clusters in preschoolers that comply or do not comply with sleep recommendations, and to investigate the association of these clusters with obesity markers. This information is critical to further understand in detail how movement behaviours are grouped in preschoolers, which may support the inception of strategies to effectively intervene and ameliorate health-related risks.

## 2. Materials and Methods

### 2.1. Setting

This study is part of the “Movement’s Cool” project, which analyses the associations between movement behaviours and health outcomes in low-income preschool children from Early Education Childhood Centers (EECC) in João Pessoa, Brazil. This study was conducted during November and December 2019; and February and March 2020, and previously approved by the Ethics Committee (protocol number: 2.727.698). The Helsinki Declarations’ ethical aspects were followed [28].

The public school system is distributed in nine educational zones of the city, where the EECC are located. These EECC are spaces for full-time education for children from 0 to 5 years old, with similar physical structures. From those, six educational zones have registered children aged between 3 and 5 years old, and three EECC, situated in deprived areas of the three different education districts, were conveniently selected. In these areas, it was observed that 62.5% of mothers or fathers were unemployed. Forty-five percent of mothers and 54% of fathers had finished the 9th grade or less.

### 2.2. Participants

In the three EECC assessed, 322 preschoolers were registered, and all of them were invited to participate in the study. Preschoolers were eligible for the study when parents or guardians signed the consent form and they did not present physical or psychological illness reported by parents, which made it impossible to participate in the study. Preschoolers were discontinued from the study when they refused to use the accelerometer or declined to participate in the study.

Thus, 28 parents or guardians did not sign the consent form, and 294 preschoolers were considered for the sample, but 22 preschoolers did not validate the minimum accelerometer wear time (8 h/day), and 272 (138 boys) completed the entire study protocol.

### 2.3. Sample Size

A posteriori sample calculation was performed, using the F tests category (ANOVA one-way), and the mean values of BMI, WC and “n” of each cluster. Thus, effect sizes for BMI (0.462) and for WC (0.506) were calculated. An alpha error of 5% and a beta error of 95% were adopted, besides the number of cluster groups. In this sense, 112 (for BMI) or 96 (for WC) preschoolers were calculated to compose the final sample, with both effect sizes corresponding to 96% of actual power.

### 2.4. Variables

#### 2.4.1. Physical Activity and Sedentary Behaviour

PA and SB were objectively assessed using accelerometers (Actigraph, model WGT3-X, Pensacola, FL, USA). Parents and preschool teachers were instructed (verbally and written) on the adequate use of the device. Accelerometers were located on the right hip for seven consecutive days (Wednesday morning to Tuesday afternoon). In addition, parents and teachers were also instructed to remove children’s accelerometers for water-based activities and while sleeping (at night). 

ActiLife software, version 6.13.3 (Actigraph, Pensacola, FL, USA), was used for device initialization, reduction of data, and analyses. Raw data were sampled at 90 Hz. Accelerometers were analysed as ActiGraph counts normalized for each child’s mean wear time in vector magnitude, considering a 15-s epoch length [29]. Data were reintegrated in 60-s epochs for analysis. Periods of ≥20 min of consecutive zero counts were defined as non-wear time and removed from the analysis [30]. The first day of accelerometer data was omitted from analysis to reduce the impact of subject reactivity [30]. Valid data were considered for a minimum of 8 h of wear time, during at least three days (one weekend day and two weekdays), concordant with a previous study [31]. The mean wear time was 10.9 h (SD ± 1.4 h of wear time between children).

To estimate the intensities of PA, cut-offs proposed by Butte et al. [32] were used, considering vector magnitude, as follows: (1) light physical activity (LPA) from 820 to 3907 counts per minute (cpm); (2) moderate physical activity (MPA) from 3908 to 6111 cpm; and (3) vigorous physical activity (VPA) ≥ 6112 cpm. SB was considered as ≤819 cpm. For the statistical analysis, LPA, moderate-to-vigorous physical activity (MVPA), and SB were considered. Then, PA categories were determined following the WHO recommendations for three to four years old [11] and five years old [33]. Thus, three- to four-year-old preschoolers who accumulated at least 180 min of PA, of which 60 min were MVPA, or five-year-old preschoolers who accumulated at least 60 min of MVPA were considered C with MVPA recommendations. Preschoolers who do not accumulate it were classified as NC (MVPA). Those who accumulated less than 180 min of PA were classified as low LPA. 

#### 2.4.2. Sleep Duration

Parents were asked about the total average hours their child slept according to the following questions: “On weekdays, how many hours of sleep does your child usually have during the night?” and “On weekend days, how many hours of sleep does your child usually have during the night?”. Overall, sleep hours were calculated as follows: ((Sleep on weekdays*5) + (Sleep on weekend days*2))/7. The results were multiplied by 60 to represent minutes per day. This method has been previously used in a similar population [34]. 

Preschoolers were classified as C or NC with sleep recommendations, according to the WHO [11] and Paruthi et al.’s [8] recommendations, being: (a) preschoolers C: aged 3 to 4 years old that sleep between 600 and 780 min/day, and five years-old that sleep 540 to 660 min/day; (b) NC: preschoolers aged 3 to 4 that sleep less than 600, and aged five years, that sleep less than 540 min/day. 

#### 2.4.3. Anthropometric Measures

A stadiometer (Holtain) and weighing scale (Seca 708, Hamburg, Germany) were used to assess height (cm) and body weight (Kg) with preschoolers being lightly dressed and barefoot. Researchers performed two measures, and if they differed, a third measure was taken. BMI was calculated as Kg/m^2^. Preschoolers’ BMI were categorized according to WHO cut-offs for this age [35]. Finally, WC was measured using a steel anthropometric measuring tape. Three measures were taken with the measuring tape positioned between the ribs and the iliac crest.

### 2.5. Statistical Analysis

Analyses for this study were performed to: (1) describe the general data for preschoolers C and NC with sleep recommendations; (2) test the correlation between variables; (3) perform the latent profile analysis, and identify the adequate number of clusters; (4) describe and compare the observed clusters; (5) associate these cluster solutions with obesity markers. Thus, these steps are described below.

First, data were tested for normality and homogeneity with Shapiro–Wilk and Levene tests. The descriptive data of the general sample of preschoolers C and NC with sleep recommendations were presented as mean and standard deviation. To compare movement behaviours and obesity markers between them, the independent samples *t*-test was adopted, with *p* < 0.05 and Cohen’s D as effect size. These analyses were performed in SPSS (20.0).

As a premise for performing the Latent Profile Analysis, the correlation between variables (SB, LPA, MVPA, sleep duration, BMI, and WC) were tested using Pearson’s correlation test and adopting r and *p*-value as the significance level. Two latent profile analyses were performed separately, to identify the number of clusters (k-classes) and class membership for preschoolers C and NC with sleep recommendations, respectively. Movement behaviours (SB, LPA, MVPA) and age in months were used for both. Models with one to six profiles were performed. The number of classes and class membership were determined using the following criteria: Bayesian information criteria (BIC), Akaike Information Criterion (AIC), entropy value (0.0–1.0), and *p*-value as the criterion of the quality of class membership classification. Thus, the cluster-solution that showed smaller values for AIC, BIC, and *p*-value was considered the better alternative. The latent profile analysis was performed in RStudio (1.3.1073; https://www.rstudio.com/ accessed on 29 December 2020), using the tidyLPA [36], MPlus Automation [37], and dplyr [38] packages. Four clusters for C preschoolers and four clusters for NC preschoolers were defined. The clusters were labelled according to their most predominant descriptive characteristics related to movement behaviours (LPA, MVPA and SB). Thus, for C preschoolers, the clusters were: C/Low SB; C/Low MVPA; C/High MVPA; and C/High LPA. For NC preschoolers, the clusters were named as: NC/Medium SB, NC/High SB; NC/High MVPA; and NC/Low MVPA.

After the clusters were defined, normality and homogeneity tests were performed (Shapiro–Wilk and Levene tests), and differences between clusters were analysed using one-way analysis of variance (ANOVA). The data were presented as mean (95%CI), adopting *p* < 0.05 and partial η^2^ as the effect size.

Finally, to test the associations between clusters, BMI, and WC, simple linear regression analyses were used, with clusters as the independent variable, considering AIC and BIC as fit model indexes. Collinearity was tested by VIF value, using SPSS (20.0) (IBM, Chicago, IL, USA) and *p* < 0.05.

## 3. Results

### 3.1. Participants’ Descriptive Characteristics According to Compliance with Sleep Recommendations

Most of the assessed preschoolers were NC with sleep and MVPA recommendations. None of the assessed children slept more than the recommended amount. Preschoolers were classified on the 50th percentile for BMI. NC preschoolers were younger (4.2 ± 0.7 vs. 4.8 ± 0.7 years old, *p* < 0.01) than their C peers (Table 1).

### 3.2. Correlations between Movement Behaviours, BMI, and WC 

Correlations were seen between SB with LPA (r = −0.18, *p* = 0.04) and sleep duration (r = −0.22, *p* = 0.01). LPA was correlated with MVPA (r = 0.42, *p* < 0.001), and sleep (r = −0.25, *p* = 0.00). Correlations between MVPA and WC were also seen (r = 0.20, *p* = 0.02), and BMI and WC were positively correlated (r = 0.60, *p* < 0.001) (Supplementary Table S1).

### 3.3. Clusters Solutions According to Compliance with Sleep Recommendations

In Table 2, four classes were observed for preschoolers C or NC with sleep recommendations, considering that these represented the best fit model to AIC, BIC, entropy, and *p*-value.

### 3.4. Graphical Representation of Clusters Solutions

**Figure 1:**
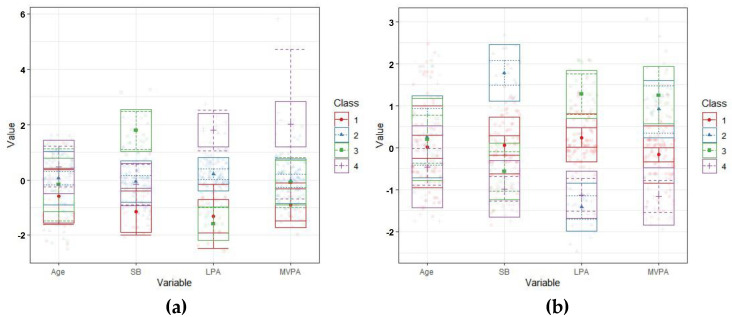
Z-score of movement behaviours clustered in four classes. (**a**) Clusters solutions of preschoolers C with sleep recommendations; (**b**) Clusters solutions of preschoolers NC with sleep recommendations.

Figure 1 presents the mean z-scores for SB, LPA, MVPA and age, for each class of preschoolers C and NC with sleep recommendations (Figure 1).

### 3.5. Descriptive Characteristics of Each Cluster Solution 

The descriptive data of each cluster solution is presented in Table 3. For C preschoolers, no significant differences in movement behaviours (SB, LPA, MVPA) were observed between clusters (*p* > 0.05), except for C/Low MVPA children (MVPA: 56.5, 95%CI: 51.9–61.0 min/day). All preschoolers were classified as normal weight, and no statistical differences were seen for BMI and WC between clusters (*p* > 0.05).

For NC preschoolers, the NC/High SB cluster had the highest SB between clusters (558.8, 95%CI: 525.9;591.7 min/day, *p* < 0.01), while the NC/Low MVPA had the lowest (278.3, 95%CI: 258.6;298.0, *p* < 0.01). NC/High SB and NC/Low MVPA clusters spent less time in LPA (139.4, 95%CI: 125.3;153.5 min/day, and 152.1, 95%CI: 140.2;164.0 min/day, *p* < 0.01). The NC/High MVPA cluster accumulated more than twice the time spent in MVPA than the NC/Low MVPA cluster (34.2, 95%CI: 28.4;39.9 min/day vs. 85.7, 95%CI: 79.7;91.6 min/day, *p* < 0.01). Concerning sleep duration, no statistical difference was observed between NC clusters (*p* > 0.05).

### 3.6. Relations between Cluster Solutions and Obesity Markers

The linear regression analysis showed that the cluster with preschoolers who comply with sleep recommendations but had low MVPA (C/Low MVPA cluster) was negatively associated with BMI (β = −0.8, 95%CI = −1.6;−0.1). However, the cluster of those who do not comply with sleep and MVPA recommendations (NC/Low MVPA cluster) was positively associated with BMI (β = 0.9, 95%CI = 0.1;1.7). No association was seen for clusters with low, medium or high SB, and no association was observed for WC (Table 4).

## 4. Discussion

The present study identified clusters of movement behaviours among preschoolers C and NC with the sleep duration recommendations, and subsequently discerned the association of these clusters with children’s BMI and WC. Although studies pertaining to preschoolers’ sleep patterns have been conducted [17,19], the present study adds important and novel information to the literature, highlighting that, according to sleep duration, preschoolers are grouped in different latent profiles, which are differently related to obesity markers. Our main findings demonstrate that the C with sleep duration recommendations and low MVPA cluster was negatively associated with BMI, while the NC with sleep duration and low MVPA cluster was positively associated with BMI. Similar results have not been seen for WC. 

Previous studies have reported that in later childhood, there is a negative association between sleep duration and BMI, as it promotes harmful alteration in satiation/appetite mechanism through subsequent downregulation [39,40]. Insufficient sleep increases fasting ghrelin level stimulating hunger, and decreases serum fasting leptin, which increases appetite, and consequently, body weight. Although these mechanisms have not been completely investigated in such young children, it is important to reinforce that preschool children should spend approximately one third of their day sleeping [8,11]. 

Vézina-In et al. [41] and Kuzik and Carson [42] showed that sleep duration is negatively associated with BMI in preschoolers. Indeed, the current results highlighted the role of sleep in BMI for those preschoolers with low MVPA. Considering the co-dependence between movement behaviours [12,43], it seems plausible that for the C preschoolers, the amount of time allocated to sleep may positively compensate the unhealthy impact of low MVPA in BMI. Moreover, unhealthy behaviours tend to aggregate [44]. Indeed, an unhealthy risk behaviour may potentiate other risk behaviours, such as short sleep and low MVPA, which negatively impact children’s health outcomes, such as BMI. Thus, understanding how movement behaviours cluster and associate with obesity markers, according to sleep duration, provides important new insights for promoting and adopting effective intervention strategies to prevent, or at least ameliorate preschoolers’ obesity.

In the present study, no association was seen for low, medium, or high SB clusters for both C and NC preschoolers. Similar results covering different analytical approaches have been previously reported in preschool children [42,45,46]. In a cluster analysis with older children, Santaliestra-Pasías et al. [21] showed that in boys, BMI, WC, and skinfolds, in clusters with greater SB (about 13 h/day) and less participation in sports (0.17 h/week), were higher than in the others, although SB had been subjectively assessed, and the role of sleep in obesity parameters had not been considered. Although the direct link between SB and BMI in youth has been previously reported [47,48], the rapid body growth observed in early childhood [49,50], combined with the intermittent movement patterns between SB and PA [51], may in somehow differentiate the observed associations, when contrasting with the results previously seen at later ages. 

Further, no difference was seen for movement behaviours or obesity markers among the four clusters of C sleepers. Conversely, NC sleeper clusters differed in movement behaviours. Based on studies conducted in adult populations, it is plausible to speculate that C preschoolers have more well-established habits throughout the day, extending to healthy waking behaviours, i.e., PA [52,53]. Nonetheless, being NC with sleep recommendations may compromise the children’s whole day, making them irritable, drowsy, or fatigued [54]. Moreover, the results also indicated that preschoolers in the NC group slept 70-to-95 min/day less than established recommendations [8], which is concordant with previous studies that have reported daily sleep deficit in preschoolers [43,55,56], and in later childhood [57]. Corroborating these results, in a systematic review, Matricciani et al. [55] reported that, during the last century, children of all age groups slept 0.75 min less each year. Indeed, the advances in technology, with overuse of television, smartphones, computers, and other types of screens, associated with the absence of sufficient boundaries of use for sleep and sleep–wake routines, may negatively affect children’s sleeping hours [20,44].

The current results did not show any association between the clusters and WC. Indeed, the association between movement behaviours with WC in this specific age group is controversial. Leppänen et al. [58] indicated no association between PA or SB with WC in 4 year-old children, while Arhab et al. [59] when assessing 2 to 6 year-old preschoolers, reported a negative association with PA and positive association with SB. Considering the co-dependence between movement behaviours, Carson et al. [60] reported no association between the 24 h composition of movement behaviours and WC.

Despite its novel and important additions to the literature, the present study has some limitations. The assessed preschoolers were normal-weight and thus, the results of the present study should be considered representative of this group. It is also important to highlight that these children spend 10 h per day at preschool settings, where they have at least three main meals, which implies in a very similar caloric ingestion among them throughout the weekday. Although it could be considered a limitation in terms of the generalisation of the results, studies are in agreement that insufficient sleep is associated with hypertension, diabetes, and mental health in adolescents, adults, and the elderly [61,62,63,64]. 

Additionally, sleep was assessed via a proxy report questionnaire answered by parents; therefore, the non-objective measure of the variable may have caused some overestimation or underestimation of the values. Despite this, children’s sleep based on parents’ reports is the most commonly used approach used in this age range, with good consistency and reliability routinely reported [20,65]. Second, we did not assess sleep quality. So, although we observed shorter sleep duration in NC preschoolers, we do not know if the quality of the sleep may have offset this. Moreover, the lack of information related to naps during the days is an important limitation, as it could be a mediating factor of total sleep duration. However, in the assessed context, children do not sleep during the day. The ECEC have the same built structure, and approximately 30 children in a small class, where the high temperature all year round, the absence of air conditioner, and the considerable noise during daytime make taking naps difficult. Despite these limitations, we were able to establish clusters for PA and SB according to compliance with sleep recommendations in a suitable number of preschoolers. Considering that behaviours established in early childhood tend to track later in life, the early detection of possible correlates and determinants of obesity are important in a public health approach.

## 5. Conclusions

Preschoolers in the low MVPA and insufficient sleep cluster showed a positive association with BMI. In contrast, a negative association was seen for those in low MVPA but sufficient sleeping hours cluster. These results suggest that sleep can be a protective factor for BMI even for those children non-compliant with MVPA recommendations.

## Figures and Tables

**Table 1:** Children’s descriptive data.

	Total (*n* = 272)	Compliant (*n* = 134) (Mean ± SD)	Non-Compliant (*n* = 138) (Mean ± SD)	*p*	Cohen’s D	X^2^ (*p*)
Gender (male/female)	134/138	67/67	67/71	-	-	0.1 (0.8)
Age (years)	4.4 ± 0.7	4.8 ± 0.7	4.2 ± 0.7 ^1^	<0.01	0.8	-
SB (min/day)	381.4 ± 97.4	382.7 ± 94.4	380.2 ± 100.6	0.8	0.0	-
LPA (min/day)	215.5 ± 50.6	217.6 ± 49.6	213.5 ± 51.6	0.5	0.1	-
MVPA (min/day)	58.6 ± 22.4	59.5 ± 24.5	57.9 ± 20.2	0.5	0.1	-
Sleep (min/day)	565.9 ± 66.6	612.1 ± 53.1	521.1 ± 43.8 ^1^	<0.01	1.9	-
BMI (Kg/m^2^)	15.8 ± 1.6	15.8 ± 1.6	15.9 ± 1.6	0.5	−0.1	-
WC (cm)	51.3 ± 3.8	51.1 ± 3.6	51.7 ± 4.0	0.2	−0.2	-

SD: standard deviation; SB: sedentary behaviour; LPA: light physical activity; MVPA: moderate to vigorous physical activity; BMI: body mass index; WC: waist circumference. X^2^: chi-squared value. Data are presented as mean and standard deviation. ^1^ Statistical difference between compliant and non-compliant preschoolers (*t*-test to unpaired sample).

**Table 2:** Criteria to model fit of latent profile analysis.

		Compliant		
Classes	AIC	BIC	Entropy	*p* Value
1	1533.09	1556.27	1.00	
2	1514.62	1552.29	0.80	0.01
3	1499.90	1552.06	0.84	0.01
4 ^1^	1474.51	1541.16	0.78	0.01
5	1463.12	1544.26	0.87	0.01
6	1456.39	1552.02	0.85	0.03
**Non-Compliant**				
Classes	AIC	BIC	Entropy	*p* Value
1	1578.49	1601.91	1.00	
2	1550.21	1588.27	0.83	0.01
3	1533.62	1586.31	0.77	0.01
4 ^1^	1494.03	1561.36	0.86	0.01
5	1484.21	1566.18	0.77	0.02
6	1491.71	1588.31	0.45	0.89

AIC: Akaike Information Criterion; BIC: Bayesian information criteria. ^1^ Better fit model according to AIC, BIC, entropy, and *p*-value.

**Table 3:** Descriptive data of latent profile classes according to compliance with sleep recommendations.

		Compliant					
	C/Low SB (*n* = 23) (Mean (95%CI))	C/Low MVPA (*n* = 91) (Mean (95%CI))	C/High LPA (*n* = 13) (Mean (95%CI))	C/High MVPA (*n* = 7) (Mean (95%CI))	*p*	Partial η^2^	X^2^ (*p*)
Gender (M/F)	12/11	47/44	7/6	1/6	-	-	3.8 (0.3)
Age (years)	4.4 (4.1;4.7)	4.9 (4.7;5.1) ^1^	4.7 (4.4;5.0)	4.8 (4.3;5.4)	0.04	0.1	-
SB (min/day)	347.6 (299.3;395.9)	392.5 (373.3;411.6)	373.9 (324.3;423.5)	387.7 (318.6;456.8)	0.3	0.0	-
LPA (min/day)	231.2 (208.7;255.3)	209.9 (199.7;220.1)	236.8 (208.4;265.3)	235.0 (217.6;252.4)	0.3	0.1	-
MVPA (min/day)	63.1 (54.4;71.7)	56.5 (51.9;61.0)	68.2 (42.4;94.1)	71.3 (51.1;91.4)	0.6	0.0	-
Sleep (min/day)	625.9 (603.3;648.5)	611.4 (600.7;622.2)	589.7 (553.2;626.2)	616.7 (565.4;668.1)	0.3	0.0	-
BMI (Kg/m^2^)	16.4 (15.9;16.8)	15.5 (15.2;15.9)	15.6 (14.9;16.3)	16.7 (15.3;18.1)	0.2	0.1	-
WC (cm)	51.6 (50.2;52.9)	50.7 (50.0;51.5)	51.8 (49.3;54.2)	52.4 (48.9;55.9)	1.0	0.0	-
		**Non-Compliant**					
	**NC/Medium SB (*n* = 84) (Mean (95%CI)** **)**	**NC/High SB (*n* = 15) (Mean (95%CI)** **)**	**NC/High MVPA (*n* = 19) (Mean (95%CI)** **)**	**NC/Low MVPA (*n* = 20) (Mean (95%CI)** **)**	***p***	**Partial** **η** **^2^**	**X^2^ (*p*)**
Gender (M/F)	38/46	9/6	12/7	8/12	-	-	3.4 (0.3)
Age (years)	4.2 (4.1;4.4)	4.4 (4.0;4.8)	4.4 (4.0;4.7)	3.9 (3.6;4.2)	0.1	0.0	-
SB (min/day)	384.7 (368.0;401.4)	558.8 (525.9;591.7) ^2^	326.4 (296.6;356.3) ^2,^^3^	278.3 (258.6;298.0) ^2,^^3^	<0.01	0.5	-
LPA (min/day)	226.3 (220.0;232.7)	139.4 (125.3;153.5) ^2^	279.9 (265.1;294.7) ^2,^^3^	152.1 (140.2;164.0) ^2,^^3^	<0.01	0.7	-
MVPA (min/day)	54.0 (51.2;56.7)	76.2 (66.0;86.3) ^2^	85.7 (79.7;91.6) ^2^	34.2 (28.4;39.9) ^2,^^3^^,4^	<0.01	0.6	-
Sleep (min/day)	526.3 (516.8;535.8)	505.4 (484.4;526.5)	506.3 (482.0;530.0)	524.8 (506.0;543.4)	0.1	0.0	-
BMI (Kg/m^2^)	15.7 (15.4;16.0)	16.0 (15.3;16.7)	16.0 (15.4;16.7)	16.5 (15.4;17.7)	0.2	0.0	-
WC (cm)	51.5 (50.8;52.3)	51.6 (49.9;53.3)	52.0 (49.4;54.6)	52.0 (49.9;54.2)	0.9	0.0	-

C: compliance with sleep recommendations; NC: non-compliance with sleep recommendations; SD: sleep duration; SB: sedentary behaviour; LPA: light physical activity; MVPA: moderate to vigorous physical activity; BMI: body mass index, WC: waist circumference; X^2^: chi-squared value. ^1^ Statistical difference with C/Low SB cluster. ^2^ Statistical difference with NC/Medium SB cluster. ^3^ Statistical difference with NC/High SB; ^4^ Statistical difference with NC/High MVPA. One-way ANOVA test.

**Table 4:** Associations between clusters and obesity markers, according to compliance with sleep recommendations.

Compliant				
BMI (Kg/m^2^)	β	95%CI	t	*p*
C/Low SB (*n* = 23)	Ref			
C/Low MVPA (*n* = 91)	−0.8	−1.6;−0.1	−2.1	0.0 ^1^
C/High LPA (*n* = 13)	−0.8	−1.9;0.4	−1.3	0.2
C/High MVPA (*n* = 7)	0.3	−1.1;1.7	0.5	0.6
**WC (cm)**	**β**	**95%CI**	**t**	***p***
C/Low SB (*n* = 23)	Ref			
C/Low MVPA (*n* = 91)	−1.4	−3.0;0.2	−1.7	0.1
C/High LPA (*n* = 13)	−0.6	−3.0;1.8	−0.5	0.6
C/High MVPA (*n* = 7)	0.6	−2.4;3.5	0.4	0.7
**Non-Compliant**				
**BMI (Kg/m^2^)**	**β**	**95%CI**	**t**	***p***
NC/medium SB (*n* = 84)	Ref			
NC/High SB (*n* = 15)	0.3	−0.6;1.2	0.7	0.5
NC/High MVPA (*n* = 19)	0.3	−0.5;1.2	0.8	0.4
NC/Low MVPA (*n* = 20)	0.9	0.1;1.7	2.1	0.0 ^1^
**WC (cm)**	**β**	**95%CI**	**t**	***p***
NC/Medium SB (*n* = 84)	Ref			
NC/High SB (*n* = 15)	−0.2	−2.4;2.0	−0.2	0.8
NC/High MVPA (*n* = 19)	0.0	−2.0;2.0	0.0	1.0
NC/Low MVPA (*n* = 20)	0.8	−1.2;2.8	0.8	0.4

Ref: reference category; C: compliance with sleep recommendations; NC: non-compliance with sleep recommendations; SD: sleep duration; BMI: body mass index; WC: waist circumference; MVPA: moderate to vigorous physical activity; SB: sedentary behaviour. ^1^ Significant association between the cluster and BMI. Model adjusted by sex and age.

## Data Availability

The data included in the present study are available upon request from corresponding author.

## Supplementary Materials

The following are available online at www.mdpi.com/article/10.3390/ijerph18189492/s1, Table S1: Correlations between SB, LPA, MVPA, sleep duration, BMI, and WC.
