# Supplementary material for: Associations between Movement Behaviours and Obesity Markers among Preschoolers Compliant and Non-Compliant with Sleep Duration: A Latent Profile Analysis
Source: Int J Environ Res Public Health. 2021 Sep 8;18(18):9492. doi: 10.3390/ijerph18189492 (PMC8472778; doi:10.3390/ijerph18189492)
Supplement: Supplementary file 1 [file ijerph-18-09492-s001.zip › ijerph-1358229-supplementary.pdf]

Supplementary file S1. Correlations between SB, LPA, MVPA, sleep duration, BMI, and WC

	**SB (min/day)**	**LPA (min/day)**	**MVPA (min/day)**	**Sleep (min/day)**	**BMI (Kg/m^2)**	**WC (cm)**
**SB (min/day)**	1	-0.18(0.04) [1]	0.09(0.14)	-0.22(0.01) [1]	-0.09(0.14)	-0.014(0.82)
**LPA (min/day)**	-0.18(0.04)	1	0.42(<0.001)[1]	-0.25(0.00) [1]	-0.09(0.16)	0.05(0.44)
**MVPA (min/day)**	0.09(0.14)	0.42(<0.001) [1]	1	-0.05(0.46)	0.04(0.47)	0.20(0.02)[1]
**Sleep (min/day)**	-0.22(0.01)	-0.25(0.00)	-0.05(0.46)	1	0.00(0.97)	-0.08(0.19)
**BMI (Kg/m^2)**	-0.09(0.14)	-0.09(0.16)	0.04(0.47)	0.00(0.97)	1	0.60(<0.001)
**WC (cm)**	-0.014(0.82)	0.05(0.44)	0.20(0.02)	-0.08(0.19)	0.60(<0.001)[3]	1

SB: sedentary behaviour; LPA: light physical activity; MVPA: moderate to vigorous physical activity; BMI: body mass index, WC: waist circumference. [1]Sgnificant correlation with LPA; [2]Significant correlation with MVPA; [3]Significant correlation with WC.
